# Sequencing of complete mitochondrial genome of brown algal *Saccharina* sp. ye-C2

**DOI:** 10.1080/23802359.2015.1137800

**Published:** 2016-02-01

**Authors:** Xiao Fan, Le Xu, Shuai Wang, Dong Xu, Xiaowen Zhang, Naihao Ye

**Affiliations:** a Yellow Sea Fisheries Research Institute, Chinese Academy of Fishery Sciences, Qingdao, China;; b Function Laboratory for Marine Fisheries Science and Food Production Processes, Qingdao National Laboratory for for Marine Science and Technology, China

**Keywords:** Complete mitochondrial genome, illumina sequencing, *Saccharina* sp. ye-C2

## Abstract

The complete sequence (37 657 bp) of the mitochondrial DNA (mtDNA) of the *Saccharina* sp. ye-C2 was determined. About 38 protein-coding genes (PCG), 3 ribosomal RNAs (rRNA) and 25 transfer RNA (tRNA) genes were annotated in the genome. The phylogenetic analysis strongly supports the close phylogenetic affinity of *Saccharina* sp. ye-C2 and *Saccharina japonica* based on the mitochondrial genomes of other brown algae.

The kelp *Saccharina* are large seaweeds (algae) belonging to the brown algae (Phaeophyceae), which contain some important economic seaweeds extensively cultivated in China, Japan and Korea (Wang et al. 2013). However, close breeding for generations in China caused loss of genetic variance in *S. japonica* (Zhan et al. 2012). Thankfully, the genome of *S. japonica* has been sequenced (Ye et al. 2015), which would powerfully push the genetic improvement of the species. Species and strains of *Saccharina* all over the world were resequenced in the project mentioned above. Enough Illumina sequencing data were provided to develop more genetic tools for *Saccharina*. In this study, the complete mitochondrial genome of a wild strain (NO. KT336420), sampled in Liaoning Province and named *Saccharina sp*. ye-C2 based on the phylogenetic analysis with 16 complete brown algae mitochondrial genomes.

The length of complete of *Saccharina* sp. ye-C2 is 37 657 bp and the genome contains 38 protein-coding genes (*rps2-4*, *rps7-8*, *rps10-14*, *rps19, atp6*, *atp8*, *atp9*, *cox1-3*, *nad1-7*, *nad9*, *nad11*, *nad4L*, *rpl2*, *rpl5*, *rpl6*, *rpl14*, *rpl16*, *rpl31*, *ORF41*, *ORF130*, *ORF377*, *tatC* and *cob*), 25 transfer RNA (tRNA) genes and 3 ribosomal RNA (rRNA) genes (*5S* rRNA, *16S* rRNA and *23S* rRNA). All 38 protein-coding genes (PCGs) have typical initiation codons (ATG). The numbers of PCGs that have complete termination codons TAA, TAG, TGA are 26, 8 and 4, respectively. Nucleotide frequency of the H-strand is as follows: T, 36.29%; A, 28.41%; C, 14.72% and G, 20.58%. The mitogenome of *Saccharina* sp. ye-C2 encodes 9637 amino acids, excluding the stop codons. All the 25 typical tRNAs, ranging from 71 to 88, possess a complete clover leaf secondary structure. The rRNAs of the *5S* rRNA, *16S* rRNA and *23S* rRNA genes are 133, 1535 and 2745 bp in length, respectively.

Phylogenetic analysis based on other 16 brown algae complete mitochondrial sequence data show that *Saccharina* sp. ye-C2 belongs to a *Saccharina* clade and are closely related to *S. japonica* (Figure 1). This result was consistent with recent phylogenetic analyses and certain morphological characters (Zhang et al. 2013; Guan et al. 2014).

**Figure 1.**
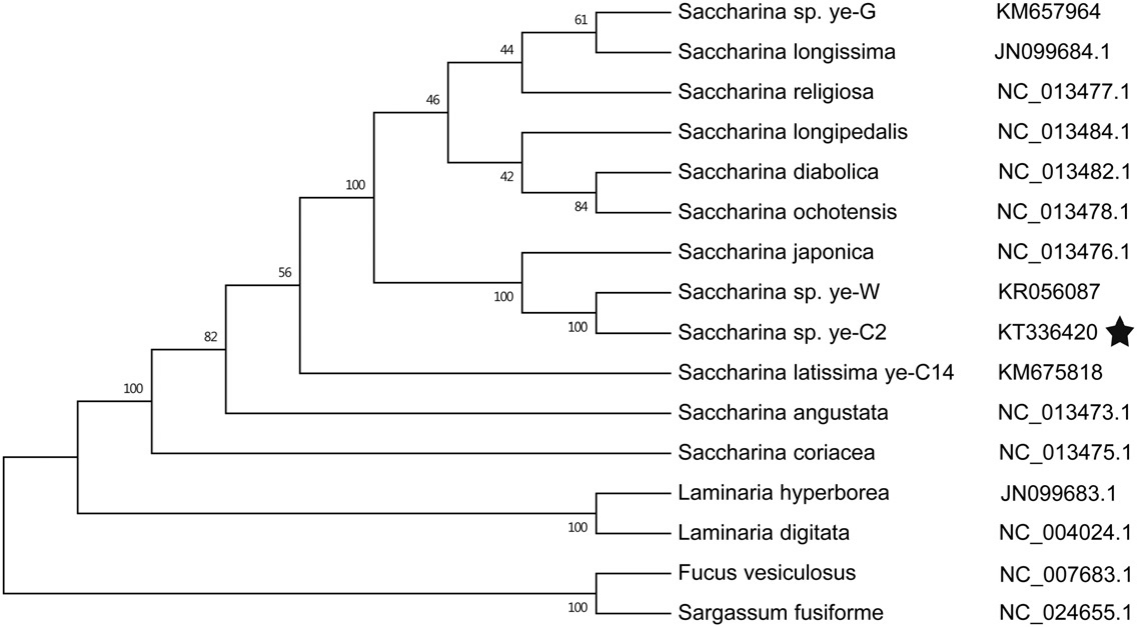
Phylogenetic tree of ML analyses based on complete mitochondrial nucleotide acid sequences of brown algae. Pentagrams stand for the species studied in this work.
